# Green Synthesis of Multi-Walled Carbon Nanotube-Reinforced Hydroxyapatite Doped with Silver and Silver-Core Selenium-Shell Nanoparticles: Synthesis, Characterization, and Biological Activity

**DOI:** 10.3390/nano15030179

**Published:** 2025-01-23

**Authors:** İlkay Unal

**Affiliations:** Department of Gastronomy and Culinary Arts, Faculty of Fine Arts, Design and Architecture Education, Munzur University, 62000 Tunceli, Turkey; ilkayunal@munzur.edu.tr

**Keywords:** bioceramics, green synthesis, hydroxyapatite, nanocomposites, silver nanoparticles, selenium nanoparticles, antimicrobial activity

## Abstract

Hydroxyapatite (HAp) is widely used in biomedical applications due to its biocompatibility, osteoconductivity, and bioactivity. However, its low mechanical strength, tendency toward rapid corrosion, and lack of bactericidal properties present significant limitations in applications. This study aimed to improve the properties of HAp by reinforcing it with multi-walled carbon nanotubes (MWCNTs) and doping it with silver nanoparticles (AgNPs) and silver-core selenium-shell nanoparticles (Ag@SeNPs). *Ocimum basilicum* extract was used as both a reducing and stabilizing agent in the synthesis of nanoparticles using an environmentally friendly and non-toxic method as an alternative to traditional methods. The synthesized HAp, HAp/MWCNT, Ag-HAp/MWCNT, and Ag@Se-HAp/MWCNT nanocomposites were characterized by TEM, SEM, XRD, Raman spectroscopy, and BET analysis. BET analysis showed a reduction in surface area from 109.4 m^2^/g for pure HAp to 71.4 m^2^/g, 47.5 m^2^/g, and 35.3 m^2^/g for HAp/MWCNTs, Ag- HAp/MWCNTs, and Ag@Se-HAp/MWCNTs, respectively. Antimicrobial activities against *P. aeruginosa*, *E. coli*, *S. aureus*, *E. faecalis*, and *C. albicans* were evaluated. HAp and HAp/MWCNT did not show any antimicrobial activity, while Ag-HAp/MWCNTs showed inhibition zones of 14 mm for *Escherichia coli* and 18 mm for *Pseudomonas aeruginosa* at 5 mg/mL. Ag@Se-MWCNTs/HAp exhibited superior efficacy with inhibition zones of 18 mm, 12 mm, and 20 mm for *S. aureus*, *E. faecalis*, and *Candida albicans*, respectively. The incorporation of Ag@SeNPs enhanced HAp’s antibacterial and antifungal properties through a synergistic mechanism.

## 1. Introduction

Each year, more than 2.2 million people worldwide require bone graft surgery to repair defects caused by diseases such as accidents, trauma, or tumor resection. The treatment of such defects is one of the major problems in orthopedic surgery [1]. Autografts, allografts, synthetic grafts, and biomaterials are the materials used for the repair of bone defects. HA, a biomaterial with the chemical formula Ca_5_(PO_4_)_3_(OH), is the major mineral component of mammalian bones and teeth [2]. HAp is widely used in biomedical applications such as bone grafts and implants, dental restorations and coatings, orthopedic implant coatings, controlled drug delivery systems, tissue engineering scaffolds, regenerative medicine, craniofacial reconstruction, and spine surgery [3,4].

Despite its widespread use and benefits, there are some limitations in the application of HAp. Its low mechanical strength, rapid corrosion, and lack of inherent antibacterial properties limit its effectiveness in medical applications, particularly in infection-prone environments like implants and prostheses. Recent research has focused on overcoming these limitations through the reinforcement and functionalization of HAp with advanced nanomaterials [5,6,7,8,9]. MWCNTs have gained attention as reinforcements due to their exceptional mechanical properties, including high tensile strength, a high elastic modulus, and similarity to collagen fibers in bone [10,11,12,13,14,15]. Studies have shown that the incorporation of MWCNTs into HAp improves the mechanical performance of the composite, densifies its microstructure, and promotes osteoblast proliferation [15]. The functionalization of MWCNTs by chemical or physical surface modifications further improves their dispersion and interactions within the HAp matrix [16].

Nanotechnology is increasingly used in many areas, including tissue engineering, drug delivery, diagnostics, imaging, and preventing bacterial infection [17]. Metal nanoparticle-based antibacterial composite materials have attracted much attention in recent years. Many studies have shown that metal nanoparticles can be effective against antibiotic-resistant bacteria. AgNPs are well known for their broad-spectrum antibacterial activity against Gram-positive and Gram-negative bacteria, as well as antibiotic-resistant strains. AgNPs incorporated into HAp-based composites effectively prevent bacterial colonization and biofilm formation, significantly reducing the risk of postoperative infections [18,19,20]. Similarly, SeNPs exhibit anti-cancer properties by inducing cell death and inhibiting the proliferation of various cancer cell types. SeNPs effectively neutralize free radicals and reduce oxidative stress with strong antioxidant capabilities, while also exhibiting antimicrobial activity against bacteria, fungi, and certain viruses [14,21,22]. The composite of AgNPs and SeNPs exhibits enhanced properties through the synergistic integration of their different properties at the nanoscale. Mostafa et al. (2023) reported that Mustafa et al. (2023) reported that Ag@Se nanocomposites exhibited high performance at low concentrations in terms of both antibacterial and antibiofilm effects [23]. A study reported that pure HAp showed no significant antibacterial activity against *Staphylococcus aureus* (Gram-positive) and *Escherichia coli* (Gram-negative), whereas Zn-HAp composites exhibited strong antibacterial effects [24]. Nanoparticles can be synthesized by chemical, physical, and biological methods, each with distinct advantages and limitations. Among these, biological synthesis methods have attracted significant attention due to their eco-friendly, cost-effective, and sustainable nature [25]. Biological methods for synthesizing nanoparticles use plants, fungi, and bacteria. Plant-based nanoparticle synthesis, often referred to as “green synthesis”, uses plant extracts containing bioactive compounds such as alkaloids, flavonoids, terpenoids and tannins, which act as natural reducing and stabilizing agents. Green synthesis has significant advantages over traditional methods, including reduced environmental impact, biocompatibility, low toxicity, and cost effectiveness [26,27,28]. The main objectives of this study are to synthesize, characterize, and evaluate the biological activities of an innovative composite material consisting of HAp reinforced with MWCNTs and doped with AgNPs and silver core Ag@SeNPs. *Ocimum basilicum* extract was used for the reduction and stabilization of nanoparticles. Finally, the antimicrobial activities of the composite materials against *P. aeruginosa*, *E. coli*, *S. aureusi E. faecalis*, and *C. albicans* were evaluated.

## 2. Materials and Methods

### 2.1. Materials

The pristine multi-walled carbon nanotubes (MWCNTs) (50–100 nm, 95% purity) were purchased from Skyspring Nanomaterials (Houston, TX, USA). Ammonium diphosphate ((NH_4_)H_2_PO_4_), nitric acid (HNO_3_, 65%), sulfuric acid (H_2_SO_4_, 95%), calcium nitrate tetrahydrate (Ca(NO_3_)_2_·4H_2_O), silver nitrate (AgNO_3_, 99.8%), Sodium selenite (Na_2_SeO_3_, 99%), Ammonia (NH_4_, 99.98%), and ethanol were purchased from Sigma-Aldrich (St. Louis, MO, USA). The plant was purchased from the local market.

### 2.2. Methods

#### 2.2.1. Preparation of Functional MWCNTs

Pristine MWCNTs were functionalized using a chemical method [29]. A solution was prepared by mixing 4.0 M HNO_3_ and 10.0 M H_2_SO_4_ in a 1:3 *v*/*v* ratio. A total of 250 mg MWCNTs was added into the solution. It was stirred vigorously at room temperature on a magnetic stirrer for 18 h. Then, it was sonicated for half an hour in an ultrasonic bath. The solution was centrifuged at 5000 rpm. The pellet was washed several times with 1/9 nitric acid solution. The oxidized MWCNTs were then washed with distilled water until the pH of the washing water reached 7 to remove residual acids. f-MWCNT was dried in an oven at 100 °C for 24 h.

#### 2.2.2. Synthesis Hydroxyapatite

Hydroxyapatite was synthesized using a simple chemical procedure. Solutions of 0.05 M Ca_2_(NO_3_)·4H_2_O and 0.03 M NH_4_H_2_PO_4_ were prepared in distilled water in separate beakers, maintaining a Ca/P ratio of 1.67. The ammonium dihydrogen phosphate solution was slowly added to the calcium nitrate solution with constant stirring at 50 °C. For hydroxyapatite formation, the pH of the mixture was adjusted to around 9–10 with 3 M NH_4_OH. After stirring for 3 h, it was aged at room temperature overnight to improve the crystallinity of the hydroxyapatite. The precipitate was filtered and washed several times with distilled water to remove unreacted precursors and byproducts. The filtered precipitate was dried in an oven at 80 °C for 24 h. Dried hydroxyapatite was calcined in a muffle furnace at 250 °C [30].

#### 2.2.3. Synthesis of MWCNT-Reinforced Hydroxyapatite

Firstly, f-MWCNTs (5 wt%) were added to an aqueous solution of 0.94 M 50 mL (Ca(NO_3_)_2_4H_2_O. It was sonicated in an ultrasonic bath for 1 h to homogenize the mixture. Then, 50 mL of 0.56 M aqueous (NH_4_)H_2_PO_4_ solution was slowly added to the mixture. After adjusting the pH of the solution to 10 using NH_4_OH, it was stirred at 50 °C for 3 h [31]. Positively charged calcium ions accumulated on the surface of the MWCNTs and combined with negatively charged phosphate ions to form calcium phosphate. It was aged at room temperature overnight. The precipitate was filtered and washed several times with distilled water to remove unreacted precursors and byproducts. The filtered precipitate was dried in an oven at 80 °C for 24 h. Dried HAp was calcined in a muffle furnace at 250 °C for 2 h.

#### 2.2.4. Preparation of Plant Extract

Four grams of the *Ocimum basilicum* plant was weighed and added to a beaker with 400 mL distilled water. It was mixed at 200 rpm for 24 h using a magnetic stirrer. The extract was filtered using filter paper and used immediately for synthesis.

#### 2.2.5. Biosynthesis of AgNP- and Ag@SeNP-Doped HAp/MWCNT Nanocomposite

Synthesis of AgNPs-doped HAp/MWCNT: Initially, 25 mL of *Ocimum basilicum* extract was put into a beaker and 0.4 g of HAp/MWCNTs was added into it. The mixture was stirred for 15 min. Then, 50 mL of 0.05 M AgNO₃ solution was added dropwise. The mixture was stirred at 200 rpm for 24 h. At the end of this period, it was centrifuged at 5000 rpm for 10 min and the precipitate was dried in an oven at 80 °C for 24 h.

**Figure 1:**
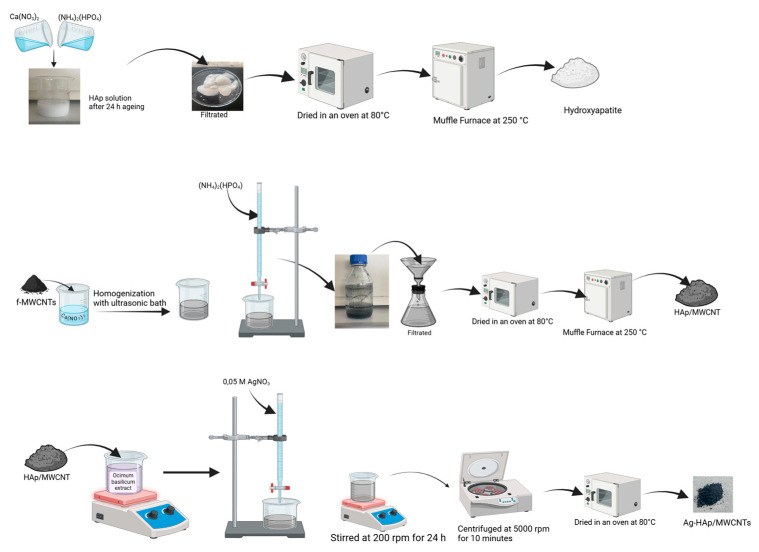
Schematic representation of the synthesis of MWCNTs reinforced Hydroxyapatite Doped with NPs.

Synthesis of Silver-Core Selenium-Shell NP-doped HAp/MWCNTs: Ag-core Se-shell NPs were synthesized with some modifications to the protocol described by Olawale, Ariatti, and Singh (2021) [22]. The initial steps were identical to the AgNP synthesis. After adding 50 mL 0.05 M AgNO_3_, the mixture was stirred for one hour to allow the formation of the silver core. Then, 50 mL 0.05 M Na_2_SeO_3_ solution was added dropwise, and the mixture was stirred at 200 rpm for 24 h. After centrifugation, the precipitate was dried in an oven at 80 °C for 24 h. The synthesis steps are shown schematically in Figure 1.

### 2.3. Characterization

X-ray diffraction (XRD) measurements were performed using PANalytical Empyrean (Holland), across a range of 2θ angles from 20° to 80°. TEM images were obtained using a Hitachi HT-7700. Micro-Raman Spectrometry was recorded using WITech alpha 300R (Germany) in the wavelength range of 350–1050 nm. Scanning electron microscopy (SEM) and energy-dispersive X-ray (EDX) analysis were performed using a MANTA 400-F (Japanese) field emission scanning electron microscope. Brunauer–Emmett–Teller (BET) surface area analysis was used for porosity determination, and the nitrogen adsorption/desorption isotherms were recorded at 77 K in the relative pressure range p/p_0_ = 0.005–1.0, using a NOVA2200e Gas Sorption Analyzer (Quantachrome, Boynton Beach, FL, USA).

### 2.4. Antimicrobial Test

The antimicrobial activity of HAp, HAp/MWCNT, Ag-HAp/MWCNT, and Ag@Se-HAp/MWCNT nanocomposites was evaluated using the agar well diffusion method on Gram-negative (*Escherichia coli* (ATCC 25922), *Pseudomonas aeruginosa* (ATCC 15442)), Gram-positive (*Staphylococcus aureus* (ATCC 25923), *Enterococcus faecalis* (ATCC. 29212)), and the fungus *Candida albicans* (ATCC 90028). Gram-positive and Gram-negative bacteria were incubated overnight on Tryptic Soy Agar at 37 °C, while *C. albicans* was incubated on Potato Dextrose Agar at 28 °C. Following incubation, microorganism suspensions were adjusted to 0.5 McFarland turbidity in physiological saline and seeded onto Müller–Hinton agar Petri dishes. Then, 8 mm diameter wells were drilled using a sterile cork borer. Samples were prepared at three different concentrations (0.5, 1, and 5 mg/mL) and 100 μL was loaded into the wells. Gentamicin and fluconazole solutions were used as control antibiotics against bacteria and fungi, respectively. The antimicrobial activity was determined by measuring the zone of inhibition (mm) around the sample wells using a standard steel ruler after 24 h of incubation [20].

## 3. Results

### 3.1. SEM-EDX Results

**Figure 2:**
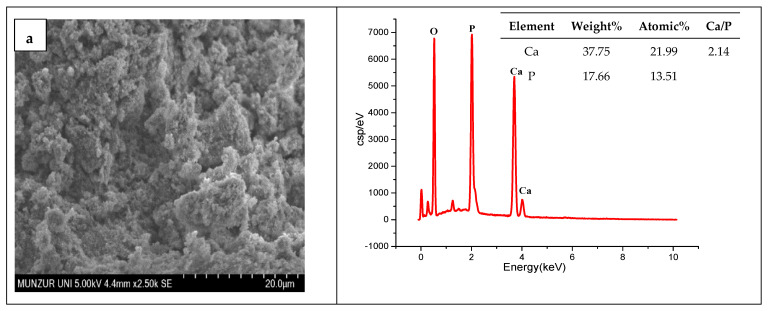

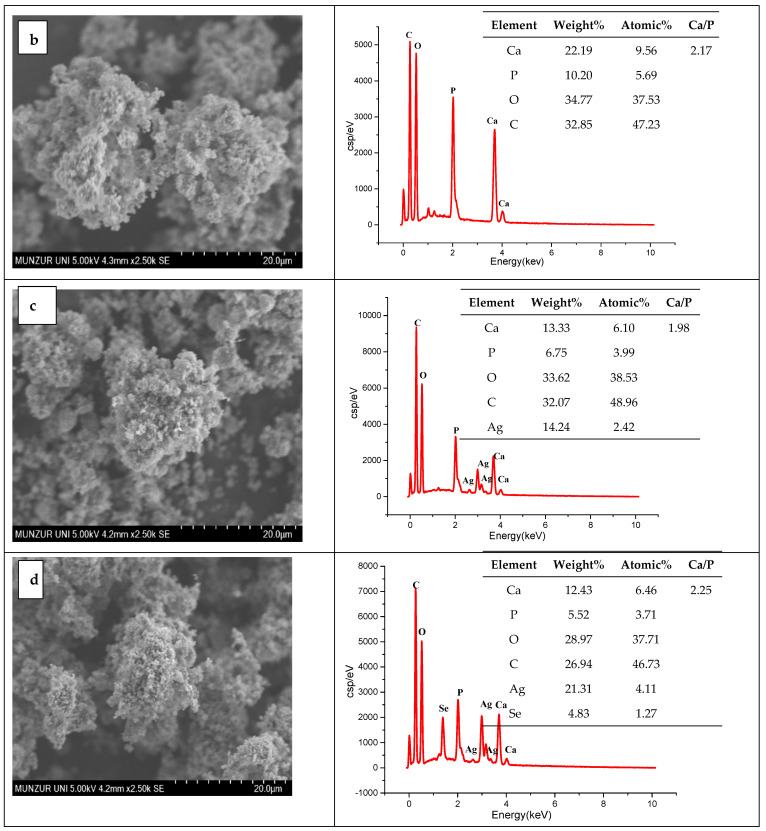
SEM images and EDX analysis reports of (**a**) HAp, (**b**) HAp/MWCNTs, (**c**) Ag-HAp/MWCNTs, and (**d**) Ag@Se-HAp/MWCNTs.

**Figure 3:**
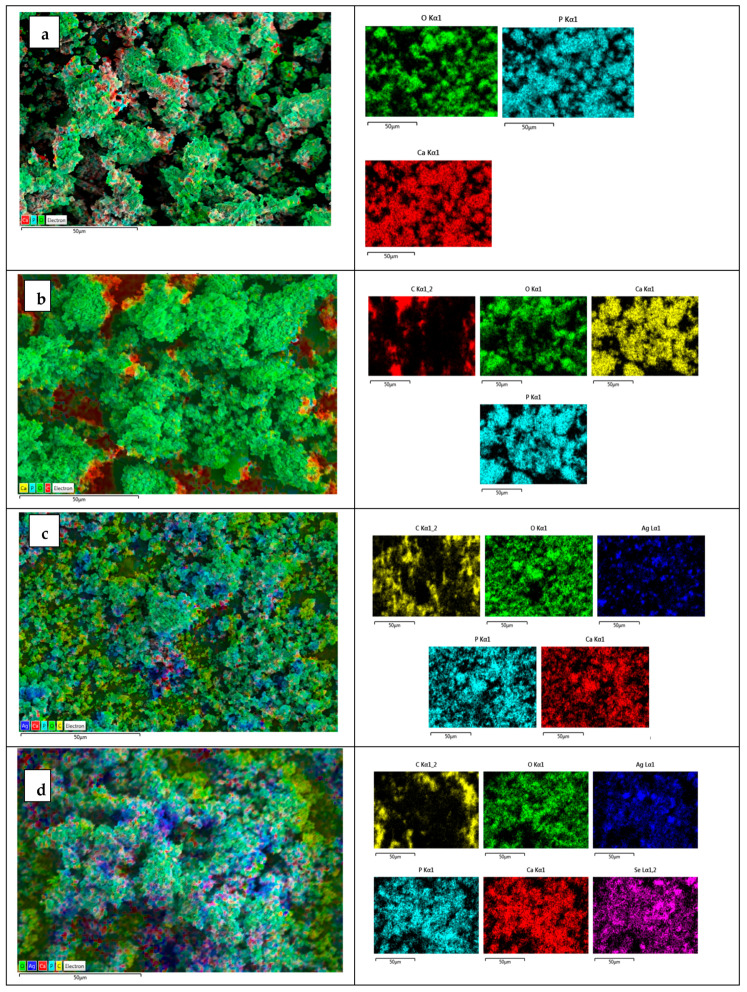
Elemental mapping of (**a**) HAp, (**b**) HAp/MWCNTs, (**c**) Ag-HAp/MWCNTs, and (**d**) Ag@Se-HAp/MWCNTs.

The structural properties and chemical compositions of the synthesized nanomaterials were analyzed via SEM and EDX analyses. The SEM analysis results showed that HAp has irregularly shaped particles aggregated into clusters. Particles with different size distributions came together and aggregated (Figure 2). MWCNTs make significant changes to the surface morphology of HAp. MWCNTs allowed for a denser and regular structure in the HAp matrix. The Ca/P weight ratio in stoichiometric hydroxyapatite is 2.15, corresponding to a molar ratio of 1.67. EDX analysis revealed that the Ca/P ratio of the synthesized HAp closely approximates the stoichiometric value. This shows that the HA produced is of high purity. The carbon peaks seen in the figure confirm the presence of MWCNT. EDX analysis confirmed the presence of Ag and Se in the HAp/MWCNT structure. While 14.285 wt% Ag was detected in the Ag-HAp/MWCNT structure, 21.31 wt% Ag and 4.83 wt% Se were found in the Ag@Se-HAp/MWCNT structure. The elemental mapping analysis shows in detail the elemental distribution in HAp composites reinforced with Ag and Ag@Se nanoparticles (Figure 3). The maps show that silver (Ag) and selenium (Se) elements are dispersed within the HAp/MWCNT matrix.

### 3.2. TEM Results

**Figure 4:**
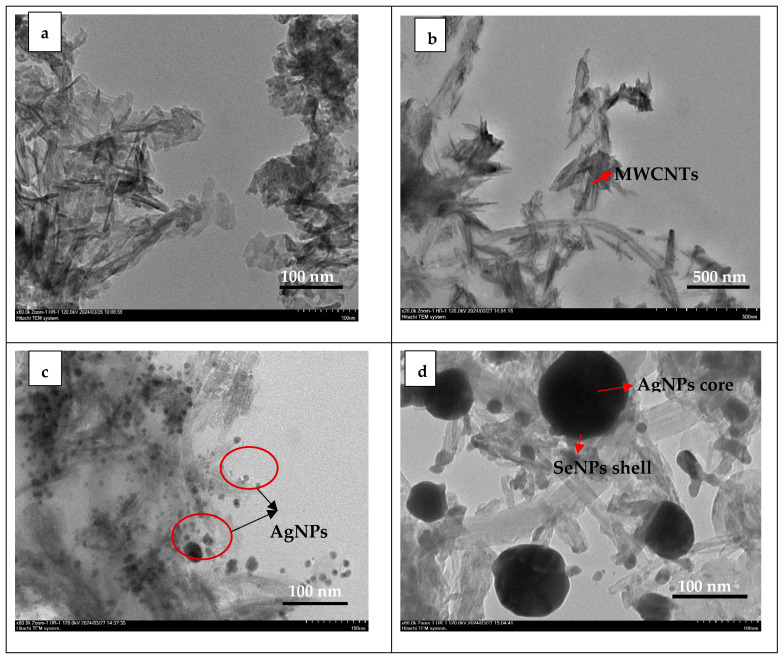
TEM images of (**a**) HAp, (**b**) HAp/MWCNTs, (**c**) Ag-HAp/MWCNTs, and (**d**) Ag@Se-HAp/MWCNTs.

TEM images of the nanocomposites are shown in Figure 4. Figure 4a shows that HAp has needle- or rod-like structures, which are common morphologies. The rods are loosely clustered, indicating some degree of aggregation that could result from van der Waals forces during the synthesis or drying processes. Figure 4b shows the successful reinforcement of f-MWCNTs into the HAp matrix and the incorporation of HAP particles on the side walls of f-MWCNTs [32]. Some deformations occurred on the surface of the nanotube functionalized by acid treatment [32,33]. In Figure 4c, it is observed that AgNPs with a spherical morphology are distributed across both the HAp matrix and the surface of MWCNTs. Ag@Se nanoparticles exhibited a spherical morphology in HAp/(Figure 4d). In the TEM images, the core and shell structures are typically distinguished by differences in density or contrast. The silver (Ag) core, with its higher atomic number and greater electron density, appears darker in the images. The size of HAp was calculated as 47.98 ± 38.3, whereas in the HAp/MWCNT structure, it was determined to be 257.42 ± 54.5, indicating that the addition of MWCNTs increases the size of the synthesized HAp. MWCNTs provide a large surface area and can act as nucleation sites, promoting the crystal growth of HAp along their surfaces, and functional groups further enhance the interaction between HAp and MWCNTs, leading to the formation of larger structures. The size of AgNPs was calculated as 47.98 ± 12.94, while that of Ag@SeNPs was determined to be 66.4 ± 38.3. The increase in size from AgNPs to Ag@SeNPs can be attributed to the formation of a selenium shell around the silver core. This core–shell structure naturally increases the overall particle size due to the additional selenium layer surrounding the silver core.

### 3.3. XRD Results

**Figure 5:**
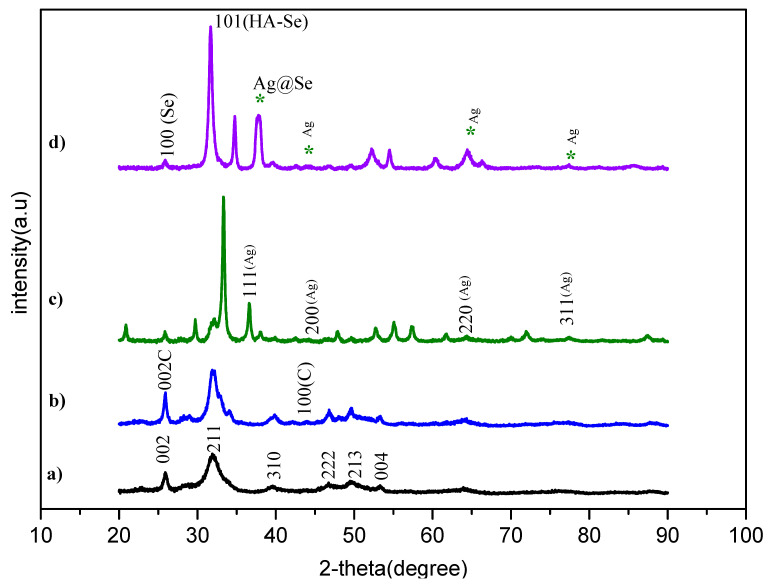
XRD graph of (**a**) HAp, (**b**) HAp/MWCNTs, (**c**) Ag-HAp/MWCNTs, and (**d**) Ag@Se-HAp/MWCNTs.

The crystal structure and phase of the samples were analyzed using XRD analysis. The XRD patterns of HAp are shown in Figure 5a. The intense peaks at 2θ = 25.9°, 21.9°, 39.5°, 46.7°, 49.8°, and 53.3° correspond to the (002), (211), (310), (222), (213), and (004) planes, which is characteristic of the hexagonal structure of HAp (JCPDS file number 9-0432) [34]. The peaks at 2θ = 25.8 and 52.9° correspond to the (002) and (100) planes, which confirm a graphitic structure (Figure 5b) (JCPDS Card No. 75-162) [35]. The XRD analysis of Ag-HAp/MWCNTa shows characteristic peaks at 2θ values of 36.6°, 42.5°, 61.7°, and 77.7°, corresponding to the (111), (200), (220), and (311) planes, respectively (Figure 5c). These peaks are consistent with the face-centered cubic (FCC) crystal structure of AgNPs (JCPDS card 04-0783) and confirm the presence of AgNPs [36]. Figure 5d shows the XRD peaks of Ag@Se-HAp/MWCNTs. The peaks are at 2θ values of approximately 225.9°, 31.6°, and 38.0°, which represent Bragg reflections in the (100), (101), and (211) planes of trigonal selenium (JCPDS File no 06-036) [37].

In the XRD graph, it can be seen that selenium peaks are weak. The matrix of HAp/MWCNTs might mask or overshadow the XRD signals of selenium. The strong XRD signals from hydroxyapatite can especially overpower the weaker selenium signals

### 3.4. Raman Spectroscopy Results

**Figure 6:**
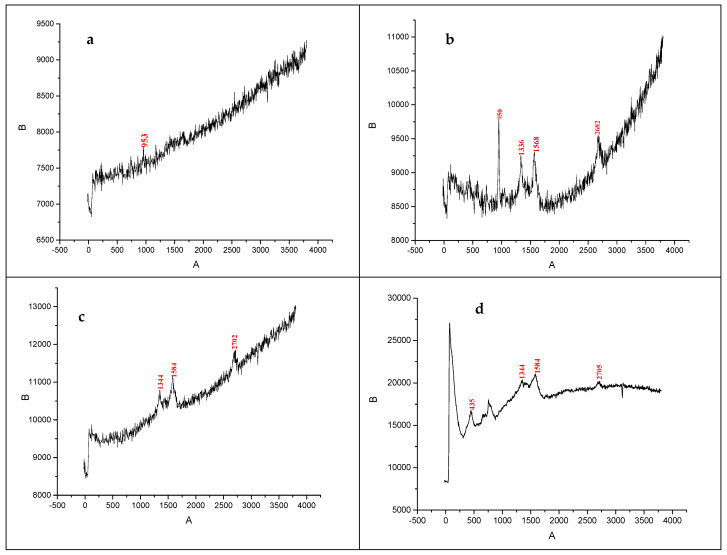
Raman spectra of (**a**) HAp, (**b**) HAp/MWCNTs, (**c**) Ag-HAp/MWCNTs, and (**d**) Ag@Se-HAp/MWCNTs.

Raman spectroscopy is a powerful technique to analyze the vibrational modes of materials. The Raman peak at 900–953 cm^−1^ is characteristic of the nanosized crystalline HAp phase, and is attributed to the symmetric stretching mode of the phosphate (PO_4_^3^⁻) group (Figure 6a) [38]. For MWCNTs, Raman peaks are typically observed at 1336 cm^−1^ (D-band, disorder-induced mode related to defects), 1568 cm^−1^ (G-band, graphite band related to the E_2_g mode of sp^2^-bonded carbon atoms), and 2682 cm^−1^ (2D-band, second-order overtone of the D-band) (Figure 6b). AgNPs typically do not show strong Raman peaks but may enhance the Raman signal of nearby molecules. The shift in the bands compared to HAp/MWCNT indicates a charge transfer mechanism of the chemical interaction between AgNPs with the surface of MWCNT (Figure 6c). Selenium peaks appear around 450 cm^−1^, but these may be weak due to the concentration and distribution of selenium (Figure 6d).

### 3.5. BET Analysis Results

BET (Brunauer–Emmett–Teller) analysis is a method used to determine the physical properties of a material, such as surface area, pore volume, and pore size. The BET analysis results of the nanocomposites are presented in Table 1. According to the BET analysis results, HAp has the highest surface area (109.4 m^2^/g) and total pore volume (0.39 cm^3^/g) and the largest pore size (14.2 nm). This indicates that HAp, with its large pores and high surface area, possesses a high adsorption capacity. The surface area (71.4 m^2^/g) and pore volume (0.19 cm^3^/g) of HAp reinforced with MWCNTs (MWCNTs/HAp) decrease significantly, while the pore size also decreases (10.8 nm). This suggests that MWCNTs integrate into the pore structure of HAp, leading to a more compact structure [39]. This change can increase the mechanical and electrical properties of the material but leads to a decrease in adsorption capacity. The addition of Ag (Ag-MWCNTs/HAp) led to a decrease in surface area and pore volume and narrowing of the average pore diameter. The further incorporation of Ag@Se (Ag@Se-MWCNTs/HAp) resulted in a continued decrease in surface area and pore volume but showed a slight increase in the average pore diameter. These changes suggest that the addition of Ag and Ag@Se progressively compressed the pore structure of the MWCNT/HAp nanocomposite. Shukla et al. (2019) reported that silver added to HAP reduced the surface area [40].

### 3.6. Antimicrobial Inhibition Zone

**Figure 7:**
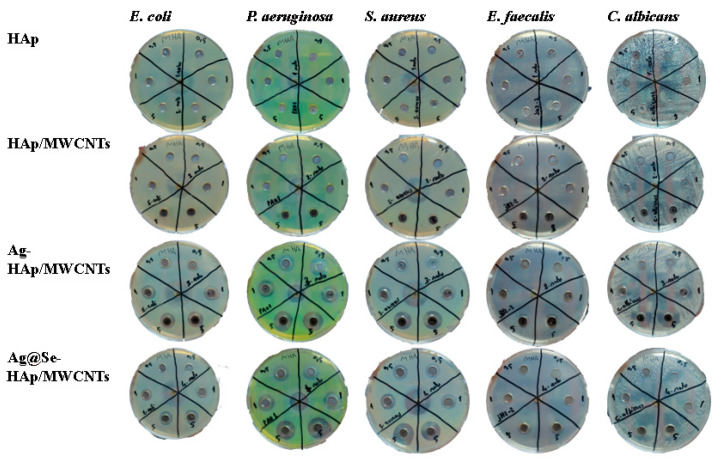
Inhibition zones of nanocomposites (0.1, 1 and 5 mg/mL).

**Figure 8:**
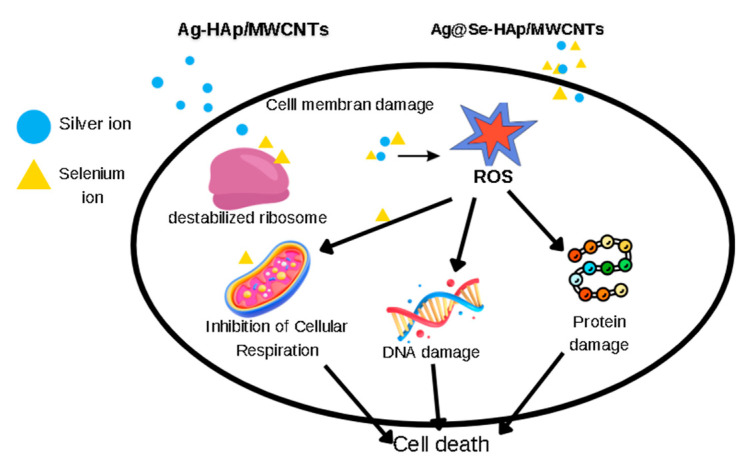
Mechanism of antibacterial activity of nanocomposites.

The antimicrobial activity of HAp, HAp/MWCNT, Ag-HAp/MWCNT, and Ag@Se-HAp/MWCNT nanocomposites was evaluated against *Escherichia coli* (*E. coli*), *Pseudomonas aeruginosa* (*P. aeruginosa*), *Staphylococcus aureus* (*S. aureus*), *Enterococcus faecalis* (*E. faecalis*), and *Candida albicans* (*C. albicans*) using the agar well diffusion method (Figure 7, Table 2). Both HAp and HAp/MWCNT nanocomposites showed no detectable zones of inhibition (ND) against any of the tested microorganisms at concentrations of 0.1, 1, and 5 mg/mL. Ag-HAp/MWCNTs demonstrated significant antimicrobial activity against *E. coli*, *P. aeruginosa*, and *S. aureus* at all tested concentrations. The zones of inhibition increased with the concentration of Ag-HAp/MWCNTs. At 5 mg/mL, the zones were 14 mm, 18 mm, and 16 mm for *E. coli*, *P. aeruginosa*, and *S. aureus*, respectively. *E. faecalis* showed a zone of inhibition only at the highest concentration (5 mg/mL). Ag@Se-HAp/MWCNTs also showed significant antimicrobial activity, with inhibition zones increasing with concentration. Ag@Se-HAp/MWCNTs. demonstrated higher antifungal activity at 5 mg/mL compared to Ag-HAp/MWCNTs. However, Ag@Se-HAp/MWCNTs showed inhibition, whereas Ag-HAp/MWCNTs did not show detectable inhibition, at 0.1 mg/mL. Coating silver nanoparticles with selenium nanoparticles increased the antibacterial and antifungal effect. Cremonini et al. (2016) reported that green-synthesized selenium nanoparticles have high antibacterial activity against the *P. aeruginosa* strain, but are less effective against *C. albicans* [41]. In contrast, the Ag@Se nanocomposite in this study was found to be more effective against *C. albicans*. SeNPs combined with AgNPs in a composite structure significantly increased antimicrobial activity. The interaction of silver and selenium ions with bacteria and fungi plays a pivotal role in the antimicrobial mechanism. Silver ions (Ag⁺) bind to bacterial and fungal cell walls, disrupting their structure and permeability. They also inhibit critical enzymes and induce oxidative stress by generating ROS, leading to cell death. Selenium ions (Se⁻ or Se^2^⁻) complement these effects by amplifying oxidative damage and interfering with the pathogen’s antioxidant defense mechanisms. While the bacterial cell wall (composed of peptidoglycan) is a primary target, fungal cell walls, which contain chitin and β-glucans, are also disrupted by these ions. Additionally, silver and selenium ions penetrate the membranes of C. albicans, causing leakage of intracellular contents and triggering apoptotic pathways [42,43]. This dual-action mechanism ensures broad-spectrum efficacy against both bacteria and fungi [44]. While Ag- HAp/MWCNTs are effective antimicrobial agents, their combination with selenium in Ag@Se-HAp/MWCNTs leads to superior performance due to enhanced ROS production and increased stability and targeting mechanisms (Figure 8). This synergistic effect leads to improved efficacy against a broader spectrum of microorganisms and more resistant pathogens [45].

## 4. Conclusions

This study demonstrates the successful synthesis and characterization of Ag- and Ag@Se-doped MWCNT-reinforced HAp composites using a green synthesis method. SEM-EDX and TEM analyses confirmed that the MWCNTs and nanoparticles were incorporated into the HAp matrix. The TEM analysis results showed that the nanoparticles have a spherical shape and that the size of Ag@Se NPs is larger compared to other nanoparticles. XRD and Raman spectroscopy also showed that the crystalline structure of HAp was retained despite the incorporation of Ag and Ag@Se nanoparticles, indicating the compatibility of these dopants with the matrix. Surface and porosity modifications were evident from the BET analysis, which showed a reduction in surface area from 109.4 m^2^/g for pure HAp to 71.4 m^2^/g, 47.5 m^2^/g, and 35.3 m^2^/g for MWCNT-HAp, Ag-HAp/MWCNTs, and Ag@Se-HAp/MWCNTs, respectively. A similar decrease in pore volume were observed, reflecting a more compact and stable composite structure. The antimicrobial evaluation of the composites highlighted their enhanced antibacterial properties. While pure HAp and HAp/MWCNTs did not exhibit significant activity, the Ag-HAp/MWCNT composite displayed inhibition zones of 14 mm for *E. coli* and 18 mm for *P. aeruginosa* at a concentration of 5 mg/mL. The Ag@Se-HAp/MWCNT composite exhibited superior antimicrobial efficacy, with inhibition zones of 18 mm, 12 mm, and 20 mm for S. *aureus*, *E. faecalis*, and *C. albicans*, respectively. Furthermore, Ag@Se-HAp/MWCNTs showed better results than AgHAp/MWCNTs in terms of antifungal activity against *C. albicans* at 0.1 and 5 mg/mL. These results emphasize the synergistic effect of silver and selenium nanoparticles, which contributed to the enhanced antibacterial performance through mechanisms such as reactive oxygen species (ROS) generation, bacterial membrane disruption, and ion release. This study highlights the potential of green-synthesized AgHAp/MWCNT and Ag@Se-HAp/MWCNT composites as next-generation biomaterials for infection control in biomedical applications. Future studies should focus on evaluating the long-term biocompatibility and mechanical properties of these composites in physiological environments using advanced characterization techniques, and investigating their in vivo performance in applications such as infection-resistant bone grafts and implants.

## Figures and Tables

**Table 1:** Results of BET analysis of nanocomposites (200 mg of each sample).

Samples	Surface Area (m^2^/g)	Pore Size (nm)	Pore Volume (cm^3^/g)
HAp	109.4	14.2	0.39
MWCNTs/HAp	71.4	10.8	0.19
Ag-MWCNTs/HAp	47.5	10.3	0.12
Ag@Se-MWCNTs/HAp	35.3	12.3	0.10

**Table 2:** Zones of inhibition (mm) of various nanocomposites against tested microorganisms.

Nanocomposite	Concentration (mg/mL)	*E. coli*	*P. aeruginosa*	*S. aureus*	*E. faecalis*	*C. albicans*
HAp	0.1	ND	ND	ND	ND	ND
	1	ND	ND	ND	ND	ND
	5	ND	ND	ND	ND	ND
HAp/MWCNTs	0.1	ND	ND	ND	ND	ND
	1	ND	ND	ND	ND	ND
	5	ND	ND	ND	ND	ND
Ag-HAp/MWCNTs	0.1	10	14	12	ND	ND
	1	13	14	14	ND	13
	5	14	18	16	11	17
Ag@Se-HAp/MWCNTs	0.1	12	14	13	ND	10
	1	12	15	13	ND	12
	5	14	18	18	12	20
Gentamicin	Control	24	15	23	11	
Fluconazole	Control					30

## Data Availability

All data from this study are available from the corresponding author upon request.
